# Toxin Levels and Profiles in Microalgae from the North-Western Adriatic Sea—15 Years of Studies on Cultured Species

**DOI:** 10.3390/md10010140

**Published:** 2012-01-17

**Authors:** Rossella Pistocchi, Franca Guerrini, Laura Pezzolesi, Manuela Riccardi, Silvana Vanucci, Patrizia Ciminiello, Carmela Dell’Aversano, Martino Forino, Ernesto Fattorusso, Luciana Tartaglione, Anna Milandri, Marinella Pompei, Monica Cangini, Silvia Pigozzi, Elena Riccardi

**Affiliations:** 1 Interdepartmental Center for Research in Environmental Sciences, University of Bologna, Via Sant’Alberto 163, Ravenna 48123, Italy; Email: franca.guerrini@unibo.it (F.G.); laura.pezzolesi@unibo.it (L.P.); manuela.riccardi@unibo.it (M.R.); 2 Department of Animal Biology and Marine Ecology, University of Messina, Salita Sperone 31, Agata, Messina 98166, Italy; Email: silvana.vanucci@unime.it; 3 Department of Chemistry of Natural Substances, University of Napoli “Federico II”, Via D. Montesano 49, Napoli 80131, Italy; Email: ciminiel@unina.it (P.C.); dellaver@unina.it (C.D.); forino@unina.it (M.F.); fattoru@unina.it (E.F.); luciana.tartaglione@unina.it (L.T.); 4 National Reference Laboratory for Marine Biotoxins, Fondazione Centro Ricerche Marine, Viale A. Vespucci 2, Cesenatico (FC) 47042, Italy; Email: anna.milandri@centroricerchemarine.it (A.M.); marinella.pompei@centroricerchemarine.it (M.P.); monica.cangini@centroricerchemarine.it (M.C.); silvia.pigozzi@centroricerchemarine.it (S.P.); elena.riccardi@centroricerchemarine.it (E.R.)

**Keywords:** Adriatic Sea, biointoxications, harmful algal blooms, toxins, ichthyotoxic species

## Abstract

The Northern Adriatic Sea is the area of the Mediterranean Sea where eutrophication and episodes related to harmful algae have occurred most frequently since the 1970s. In this area, which is highly exploited for mollusk farming, the first occurrence of human intoxication due to shellfish consumption occurred in 1989, nearly 10 years later than other countries in Europe and worldwide that had faced similar problems. Until 1997, Adriatic mollusks had been found to be contaminated mostly by diarrhetic shellfish poisoning toxins (*i.e.*, okadaic acid and dinophysistoxins) that, along with paralytic shellfish poisoning toxins (*i.e.*, saxitoxins), constitute the most common marine biotoxins. Only once, in 1994, a toxic outbreak was related to the occurrence of paralytic shellfish poisoning toxins in the Adriatic coastal waters. Moreover, in the past 15 years, the Adriatic Sea has been characterized by the presence of toxic or potentially toxic algae, not highly widespread outside Europe, such as species producing yessotoxins (*i.e.*, *Protoceratium reticulatum*, *Gonyaulax spinifera* and *Lingulodinium polyedrum*), recurrent blooms of the potentially ichthyotoxic species *Fibrocapsa japonica* and, recently, by blooms of palytoxin-like producing species of the *Ostreopsis* genus. This review is aimed at integrating monitoring data on toxin spectra and levels in mussels farmed along the coast of the Emilia-Romagna region with laboratory studies performed on the species involved in the production of those toxins; toxicity studies on toxic or potentially toxic species that have recently appeared in this area are also reviewed. Overall, reviewed data are related to: (i) the yessotoxins producing species *P. reticulatum*, *G. spinifera* and *L. polyedrum*, highlighting genetic and toxic characteristics; (ii) Adriatic strains of *Alexandrium minutum*, *Alexandrium ostenfeldii* and *Prorocentrum lima* whose toxic profiles are compared with those of strains of different geographic origins; (iii) *F. japonica* and *Ostreopsis* cf. *ovata* toxicity. Moreover, new data concerning domoic acid production by a *Pseudo*-*nitzschia multistriata* strain, toxicity investigations on a *Prorocentrum* cf. *levis*, and on presumably ichthyotoxic species, *Heterosigma akashiwo* and *Chattonella* cf. *subsalsa*, are also reported.

## Abbreviations

AA: 5,8,11,14-eicosatetraenoic acid
BTX: Brevetoxin
DA: Domoic acid
DSP: Diarrhetic shellfish poisoning
DTX: Dinophysistoxin
EC_50_: 50% effect concentration
ELA: Erythrocyte lysis assay
EPA: 5,8,11,14,17-eicosapentaenoic acid
GTX: Gonyautoxin
ITS: Internal transcribed spacer
LC-MS: Liquid chromatography-mass spectrometry
LSU: Large subunit
NEO: Neosaxitoxin
NMR: Nuclear magnetic resonance
OA: Okadaic acid
OTA: 6,9,12,15-octadecatetraenoic acid
OVTX: Ovatoxin
pPLTX: Putative palytoxin
PSP: Paralytic shellfish poisoning
PUFAs: Polyunsaturated fatty acids
ROS: Reactive oxygen species
SEM: Scanning electron microscope
SSU: Small subunit
STX: Saxitoxin
YTX: Yessotoxin

## 1. Introduction

Harmful algal blooms (HAB) have started to represent a worldwide threat to human health and/or to wild and farmed fauna since the 1970s. The most relevant intoxication episodes were linked to the production of toxins by algae belonging to the Dinophyceae class and recognized as different human syndromes: diarrhetic shellfish poisoning (DSP), paralytic shellfish poisoning (PSP), neurotoxic shellfish poisoning (NSP), and ciguatera fish poisoning (CFP). The DSP, PSP, and NSP share some common features and are primarily associated with direct consumption of filter-feeding shellfish that accumulate the algal toxins. Up to now, the toxins responsible for DSP are represented by okadaic acid and dinophysistoxins (produced by several *Dinophysis* species and *Prorocentrum lima*), while saxitoxins (produced by *Alexandrium* spp., *Gymnodinium catenatum* or *Pyrodinum bahamense*), and brevetoxins (produced by *Karenia brevis*) are those responsible for PSP and NSP, respectively. CFP is caused by consumption of tropical fish contaminated by toxins such as ciguatoxins and maitotoxins primarily attributed to the dinoflagellate *Gambierdiscus toxicus* and transferred by herbivorous fish to higher trophic levels. More recently, another shellfish syndrome, namely the amnesic shellfish poisoning (ASP), has been reported and attributed to diatoms belonging to *Pseudo-nitzschia* genus, which are domoic acid producers. The causative algal species involved in these intoxications, their ecology, their toxins’ production profiles, and their effects on human health have been extensively reviewed, as well as bloom dynamics of the ichthyotoxic species which more frequently cause severe damages to farmed fish [1,2,3]. 

**Figure 1:**
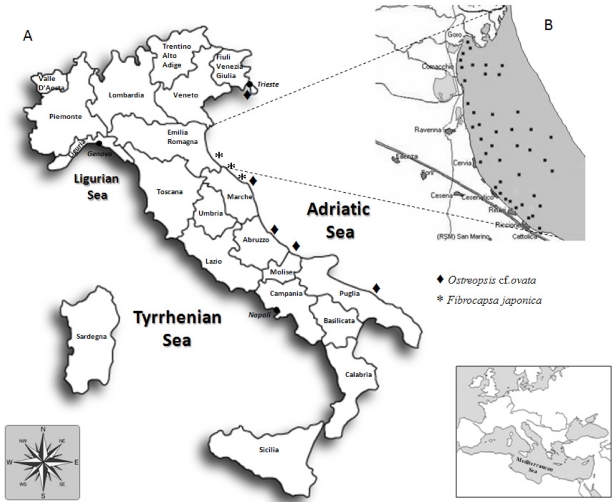
Map of Italy reporting the Italian Regions and showing (**A**) the areas of the Adriatic Sea where recurrent blooms of *Fibrocapsa japonica* and the presence of *Ostreopsis* cf. *ovata* have been detected; and (**B**) a detail of the Emilia-Romagna coast indicating (dots) locations of mussel farms from where most of the studied toxic species were detected and isolated.

The increase in HAB frequency and expansion, especially of those related to DSP and PSP diseases, have led most countries to establish regular monitoring activities for both monitoring harmful phytoplankton in coastal waters, and for assessing levels of toxins in farmed mollusks; accordingly in Italy, DSP and PSP toxin analyses in mussels started in 1976, and were extended to ASP in 1990s. Nonetheless, over the past 15 years, the Adriatic Sea (Figure 1A) has been characterized by the presence of toxic or potentially toxic algae not highly widespread outside Europe, with consequences for human or faunal health not sufficiently clarified. In fact, the main problems have been dealing with: (1) the presence of yessotoxins producing species (*i.e.*, *Protoceratium reticulatum*, *Gonyaulax spinifera* and *Lingulodinium polyedrum*) that prompted mussel farms closure for several months of many years; (2) the recurrent blooms of the potentially ichthyotoxic species *Fibrocapsa japonica* and, recently; (3) the presence of blooms of palytoxin-like producing species belonging to *Ostreopsis* genus. When first bloom episodes of these species occurred, little was known on several aspects such as their toxins’ production, structure and mechanism of action, as well as the role of the environmental conditions on toxin production. Therefore, the need to increase knowledge on potential risks for humans and ecosystem stimulated research in the field. This paper is meant to provide a report of the results, partly reviewed and partly new, of our studies on the mentioned organisms highlighting inter- and intraspecific variability of the toxin profiles and, in some cases, linking differences to algal genetic variability. In addition, we report also on toxicity of microalgae such as *Alexandrium ostenfeldii*, *Pseudo-nitzschia* spp., *Prorocentrum lima*, *Heterosigma akashiwo* and *Chattonella* cf. *subsalsa* which, so far, have never represented a concrete problem for Italian waters, as they were detected either sporadically or in low amounts; however, the characterization of their toxins can be of interest for research and for monitoring programs on aquaculture farms and coastal waters.

A complete report of all the harmful species detected, up to now, in the Adriatic Sea is beyond the scope of this paper, as is the description of blooms of the mucilage-forming species *Gonyaulax fragilis* which periodically occur as a peculiar feature of this basin [4], but have never been associated with toxic episodes. Additional information on harmful species in Adriatic Sea can be found in various other studies [5,6,7,8,9].

## 2. Overview of Toxins Detected in Shellfish Farmed along the Coast of the Emilia-Romagna Region from 1980s to Date

### 2.1. Toxins Affecting Humans Due to the Consumption of Mussels and Responsible for Shellfish Farms Closure

In Italy, the first cases of human intoxication due to fishery products occurred during the 1970s and they were caused by imported mussels and fish: in 1976, several cases of PSP occurred in people who had eaten mussels imported from Spain, while in 1977 and 1978 intoxication episodes were caused by fish imported from a tropical area and contaminated with tetrodotoxin [10]. Following these episodes and considering that the Adriatic Sea often experienced algal blooms as a consequence of heavy eutrophication, in 1978 regulatory limits for DSP and PSP toxins were introduced. Although several potentially toxic dinoflagellate species were observed in seawater samples over those years [11,12], the first intoxication event (*i.e.*, DSP) due to the presence of okadaic acid (OA) and dinophysistoxins (DTXs) [13] in mussels farmed in the North-Western Adriatic Sea (Emilia-Romagna coast; Figure 1B) occurred in 1989 and it was related to the presence of various species belonging to the genus *Dinophysis* [12]. Until 1997, mussels resulting positive to the mouse assay have been recurrently found, concurrently with the presence of *Dinophysis* spp. [14,15], although these species were usually present at low concentrations, ranging mostly between 10^3^ and 10^4^ cells/L [12]. In the following years the presence of *Dinophysis* spp. and toxins in mussels have been reported in the Adriatic Sea [8,16]. Diarrhetic poisoning appeared again in Italy in the late summer of 2010; on that occasion more than 200 people were affected due to consumption of mussels containing a high level of OA, as the only diarrhetic toxin present, alongside pectenotoxin-2 and yessotoxin (YTX) [17].

In 1995, both OA and YTX were found in mussels from the Northern Adriatic farms, and thereafter, yessotoxins represented the main contaminants of the Adriatic shellfish replacing OA and DTXs. As described below, yessotoxins are reported to be produced by dinoflagellates belonging to different genera of the order Gonyaulacales (*i.e.*, *Protoceratium reticulatum*, *Lingulodinium polyedrum*, *Gonyaulax spinifera*) and they were all associated with mussel contamination in the Northern Adriatic Sea. Until 2006, the high YTX levels usually detected in shellfish were associated to *P. reticulatum* blooms that caused farm closures for prolonged periods (up to 220 and 153 days in 2002 and 2004, respectively). In 2007, farm closures were associated with high levels of homo-YTX correlated with the presence of high densities of *G. spinifera* (up to 7.1 × 10^5^ cells/L).

As for PSP toxins, trace amounts were detected in 1993, for the first time, in mussels farmed along the Emilia-Romagna coast [18]. The responsible species was not unequivocally identified, but two *Alexandrium* species (*A. tamerense* and *A. minutum*) were reported to occur in that costal area of the Adriatic Sea [11,19,20]. The presence in mussels of a simple toxin profile dominated by gonyautoxins (GTX2 and GTX3) suggested that *A. minutum* was very likely the causative organism, as suggested by multiple lines of evidence: firstly several *A. minutum* strains previously analyzed showed the absence of toxins C1 through C4, saxitoxin (STX) and neosaxitoxin (NEO) in their toxin profile, which conversely were evidenced in some *A. tamarense* strains and, secondarily, a bloom of *A. tamarense* which occurred in 1982 was not correlated with mussel toxicity despite the presence of up to 1.8 × 10^7^ cell/L [12,21]. The only episode of mussels contaminated with PSP toxins in amounts exceeding the regulatory limit occurred in 1994, and the responsible organism was identified as *A. minutum* [22]. The high mussel toxicity was due to a large and persistent bloom (max 7.3 × 10^3^ cell/L) [23]. Successively, the presence of low levels of PSP toxins was sporadically detected in mussels ([24] and personal observations).

### 2.2. Toxins Sporadically Detected in Cultivated Mussels

Starting from the year 2000, domoic acid (DA) entered the toxic profile of the Adriatic mussels farmed both along the Italian [25,26] and Croatian coasts (Western and Eastern sides of the Northern Adriatic Sea, respectively) [9,27,28], although the detected levels were always below the regulatory limit; concurrently various *Pseudo-nitzschia* spp. blooms were observed in these coastal waters ([7,9,28] and personal observations).

Over the past decade, LC-MS-based chemical analyses of shellfish samples, performed in parallel with the regulatory mouse bioassay, allowed the detection of new toxins that were present at levels far below the regulatory limits. For example, in 2003 spirolides, a group of toxic macrocyclic imines, were detected for the first time in the North-Western Adriatic Sea, concurrently with a bloom of the dinoflagellate *Alexandrium ostenfeldii* that lasted a few months reaching a maximum cell abundance of 1.6 × 10^4^ cells/L. Later on, this species never again reached these high concentrations although traces of spirolides were sporadically detected in shellfish concurrent with the presence of few *A. ostenfeldii* cells in water samples (Milandri A., personal observations).

## 3. Algal Species and Toxins Mostly Affecting the Ecosystem and/or Humans by Aerosol Inhalation

Since the summer of 1994, the Raphidophyceae *Fibrocapsa japonica* has become the most recurrent blooming species in the Western Adriatic coastal area causing heavy water discoloration especially in the first 100–150 m from the shore (*i.e.*, the bathing zone). Blooms usually appear in patches covering some regions facing the Northern Adriatic Sea, especially Emilia-Romagna and Marche regions, with cells concentrations exceeding 10^6^ cell/L [29,30,31]. During these blooms, other Raphidophyceae (*Heterosigma akashiwo*, *Chattonella* spp.) are usually present in very low concentrations; however, these species can exceptionally form nearly monospecific blooms as occurred in July 2011 for *H. akashiwo* and *C. globosa*. Various Raphidophyceae species are reported to be ichthyotoxic, thus the appearance of these blooms caused some concern although no fish mortality was reported. 

Toxic outbreaks by *Ostreopsis* spp. occurring since the beginning of this century represent a completely new problem, affecting mainly the Mediterranean Sea [32,33,34]. It caused great concern after the 2005 episode occurring near Genoa (Liguria, Tyrrhenian Sea) when nearly 200 people needed medical care [35]. Concerning the Adriatic Sea, the situation is, to date, highly variable as the Southern Adriatic Sea was among the first zones where human suffering, correlated with *O.* cf. *ovata* bloom, were reported in 2001 [36]; in the Marche region its presence has been observed since 2006 [37,38], and in 2008 a bloom was correlated with human and animal suffering also inducing closure of the bathing area. In other Italian coastal regions (Friuli-Venezia-Giulia, Abruzzo and Molise) *O.* cf. *ovata* was detected following appropriate monitoring programs although without reporting any consequences for humans and ecosystem [39,40,41], while in two regions (Veneto and Emilia-Romagna) this species has never been found ([39] and personal observations).

## 4. Studies on Qualitative and Quantitative Toxin Profiles of Cultured Microalgae

Most of the toxic or presumably toxic species detected in the Adriatic Sea were isolated and cultured in laboratory, with the exception of *Dinophysis* spp. for which an adequate protocol for their culturing was not available until 2006. The cultured algal strains were analyzed to determine their toxin profiles, and for comparison with toxin patterns of contaminated mussels when available. When toxicity was uncertain, attempts to induce it by exposing cultures to stressful conditions were performed. Intraspecific toxic variability among strains of the same species isolated from different geographical areas was evaluated, and in some cases a genetic variability was also highlighted. This chapter summarizes the results of our studies performed in the past 15 years on cultured toxic species.

### 4.1. Yessotoxin Group

In 1995, abnormal neurotoxic effects were found in mice used for the bioassays performed for DSP shellfish monitoring programs in the Emilia-Romagna coast. Immediately afterward the effect was ascribed to the presence of a compound identified as YTX [42], a toxin previously characterized by Murata [43] and whose biogenetic origin at that time was unknown. During recurrent toxic episodes, occurring from 1997 to 1999, dinoflagellate strains were isolated from field samples and analyzed for toxicity leading to the identification of *Protoceratium reticulatum* as the organism responsible of YTX production in the Adriatic waters [44], as for the New Zealand *P. reticulatum* strain [45]. Subsequently, the *P. reticulatum* toxin profile was found to be complex and characterized by the presence of a variety of analogs, such as homoYTX, 45-OHYTX, carboxyYTX and noroxoYTX, which had been previously recorded in mussels, and therefore attributed for the first time to an algal metabolic activity [46]. Since 1997, *P. reticulatum* has represented a recurrent species in the Adriatic waters, thus several strains have been isolated and cultured. All the 11 cultured strains, isolated between 1998 and 2005, were shown to be toxic producing YTX as the main toxin. Similarly, all the *P. reticulatum* strains isolated worldwide were reported to be toxic, some of them producing only the YTX [47], while others displaying a variety of analogs [48] and/or different prominent toxins [49,50]. The several Adriatic strains showed variability in the total toxin levels, ranging between 4.5 and 65.0 pg/cell [51]; the upper levels were among the highest concentrations measured worldwide leading to high mussels toxicity, justifying prolonged closure of shellfish farms for several months, even after *P. reticulatum* disappeared in seawater samples. 

In addition, the 18S rDNA (SSU) of the above-mentioned 11 strains was sequenced [52], as well as the 28S rDNA (LSU) of three of them [53]. All the strains reported identical sequences evidencing a genetic uniformity among clones of the Adriatic Sea, as well as among these strains and strains from different geographical areas [54,55,56]. These results are reported in the phylogenetic trees [57] where Adriatic *P. reticulatum* strains are identified with the sequence access numbers DQ217789 and EF065552 for SSU and LSU, respectively. No genetic diversity was observed among coastal ocean *P. reticulatum* isolates [58], while a slight difference was found between the latter isolates and a strain from a saline lake for LSU and ITS regions of the rDNA.

Since 1997, *Lingulodinium polyedrum* has been also reported to be a producer of YTXs as inferred by homo-YTX contaminated mussels in concurrence with a nearly *L. polyedrum* monospecific bloom [59]. Different batch cultures of a *L. polyedrum* strain, isolated in our laboratory from samplings from the same area and period were non-toxic or produced low amounts of homoYTX [21], in contrast to Spanish strains which were found to produce low amounts of YTX [60]. Toxic and non-toxic strains were also reported from different areas [58,61]; however genetic analyses, performed on the investigated strains, showed very low intraspecific diversity (both in the ITS and LSU rDNA regions) despite the variability in toxicity [58]. A further investigation of our strains allowed us to find low levels of homoYTX (0.013 pg/cell), in a culture grown in f/2 medium, which increased to 0.033, 0.041 and 0.080 pg/cell by cultivating the algae with decreasing phosphorus amounts (7.26, 1.45 and 0 μM, respectively) [62]. These results evidence a high variability in the toxicity of this species or, alternatively, that *L. polyedrum* becomes toxic only under certain environmental conditions. With regard to the Adriatic area we can affirm that, from 1976 to 1984, this species sustained severe blooms causing seawater discolorations, although these events were never associated with toxic mussels [2].

A certain variability also characterizes *Gonyaulax spinifera*, identified as a YTX-producing species in 2006 [63]. This organism has also been responsible for several bloom episodes in the Adriatic Sea without any connection with mussel toxicity. However, in 2004 an unusual abundance of homoYTX detected in mussels occurred concurrently with the presence of high numbers of *G. spinifera* cells and low cell densities of *P. reticulatum* and *L. polyedrum*. This led us to investigate *G. spinifera* toxicity in more detail, as previous ELISA analysis of a New Zealand strain did not discern the main toxin produced. Two strains isolated from the Emilia-Romagna coast in 2004 and 2006 showed marked differences both in toxin profile and in genetic characteristics: the former produced YTX as main toxin, while homoYTX clearly was predominant in the latter. The two strains differed for both 18S (GenBank access number DQ867107 and EU805590) and 28S (EF416284 and EU805591) rDNA sequences, and only the 2006 strain clustered with the New Zealand isolate [57]. Subsequently, four strains isolated in 2007 were additionally analyzed and displayed 28S rDNA sequences matching those of the 2006 strain, thus again clustering with the New Zealand strain [64]; the toxin profile was not analyzed, however the presence of this species in field samples was always correlated with the presence of YTX homo-derivatives in mussels [65].

These results highlighted the high variability of *G. spinifera* or, alternatively, that the population that sustained the 2004 bloom contained cryptic species. Unfortunately the 2004 strain was viable only for a short time and further investigations, through SEM or additional molecular analyses to discriminate genetic variation within populations [66,67], could not be performed. However, the study carried out by Howard [58] on the phylogeny of YTX producing species, substantiates the latter hypothesis.

### 4.2. Saxitoxin Group

Intoxications due to PSP toxins have never represented a serious problem for the Adriatic Sea, and therefore, investigations on toxicity of *Alexandrium* species in our laboratory lagged behind those of the other toxic species. However, an *A. minutum* strain isolated in 1993 from the Gulf of Trieste (kindly provided by G. Honsell) was utilized for setting up of a new functional method for PSP toxins detection [68]. GTX4 represented nearly 98% of the total cell toxin content, followed by GTX1, GTX2 and GTX3 reporting levels of 964, 17, 3 and 2 fg/cell, respectively. These four compounds constitute the saxitoxin (STX) analogs mostly found in *A. minutum/lusitanicum* species complex [69,70], although some exceptions have been observed [71]. Toxin content was found to increase under P-limitation, as observed for other *A. minutum* strains [72,73], with GTX3 having the higher increase (nearly 12-fold compared with a 3-fold increase for GTX1 and GTX2 and 6-fold for GTX4). Thus, although GTX4 represents a toxin with low relative potency, under P-deficiency the overall toxicity of *A. minutum* populations can increase by nearly six times.

### 4.3. Okadaic Acid Group

**Figure 2:**
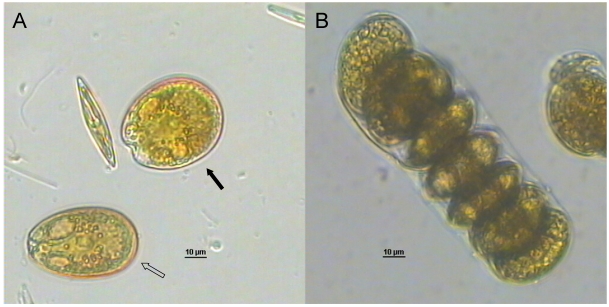
(**A**) *Prorocentrum* cf. *levis* (filled arrow) and *Prorocentrum lima* (empty arrow) cells in a field sample and (**B**) a long branching chain of adherent cells in a *P.* cf. *levis* culture.

As mentioned, various *Dinophysis* species were responsible for DSP episodes occurring since the late 1980s due to consumption of cultivated or wild mussels in the Northern Adriatic Sea. Besides *Dinophysis* spp., some species belonging to the *Prorocentrum* genus are known to produce OA and DTXs, especially globally distributed *Prorocentrum lima* which, however, has been seldom linked to human intoxication due to its benthic habitat [74]. In Spring 2002 a DSP episode occurred when people ate mussels collected from the shallow waters of a lagoon near Goro (North Emilia-Romagna coast, Figure 1B), one of the most important Italian system for aquaculture. This episode promptly led us to examine the benthic microalgae community. Taxonomic analyses carried out on water samples collected close to the bottom (~1.5 m depth) evidenced the presence of typical benthic species belonging mostly to *Prorocentrum* genus, as well as various *Dinophysis* spp. Two *Prorocentrum* strains were then isolated: one was identified as *P. lima* while the other one could not be identified. Following the identification by Faust [75] of a new species from the Belizean barrier reef system, named *P. levis*, we could refer to the above uncharacterized *Prorocentrum* strain as *P.* cf. *levis*. This identification was achieved on the basis of its morphometric characteristics (length 43–46 μm, width 37–40 μm) and cell chain formation (Figure 2), which appeared superimposable with those described by Faust for *P. levis.*

Both *P. lima* and *P.* cf. *levis* were analyzed for toxin profile; the Adriatic *P.* cf. *levis* turned out not to contain any of the known toxins, in contrast to *P. levis* isolated from Belize waters which was shown to produce OA and dinophysistoxin-2 (DTX2) [75]. Conversely, *P. lima* was found to be toxic, producing OA as the main toxin (in the range 6.7–8.4 pg/cell), DTX1 at nearly 60-fold lower levels, and DTX2, DTX4 and DTX6 in negligible amounts. The OA cell levels were within the range of values reported for most *P. lima* strains isolated worldwide [76]; whereas, the DTX1 levels (mean value: 0.12 pg/cell) were much lower than those reported for many isolates, especially for most European strains which were characterized by an OA/DTX1 ratio not higher than 4.5. 

### 4.4. Cyclic Imine Group

An *Alexandrium ostenfeldii* strain was isolated during a bloom occurring in 2003 along the Emilia-Romagna coast. In general, throughout the world this species shows high variability in its toxin profile. For example a strain from Nova Scotia (Canada) was found to contain high levels of spirolides A, B, C, D, 13-desmethylspirolide C (13-desMe C) and 13-desMeD [77], while none of these compounds were found in New Zealand strains, for which the only PSP toxin production was reported [78]. Finally, some Scandinavian isolates were reported to produce both spirolides and PSP toxins [79]. The Adriatic *A. ostenfeldii* strain was found to be toxic containing only spirolides, with 13-desMeC being the main toxin, and quantified at a level of 3.7 pg/cell [80]. Other spirolides were also recorded in low quantities from large amounts of cultures; specifically, 13,19-desMeC was identified as the second most abundant compound, and new analogs were detected and the structure elucidated by NMR [81,82].

### 4.5. Domoic Acid Group

**Figure 3:**
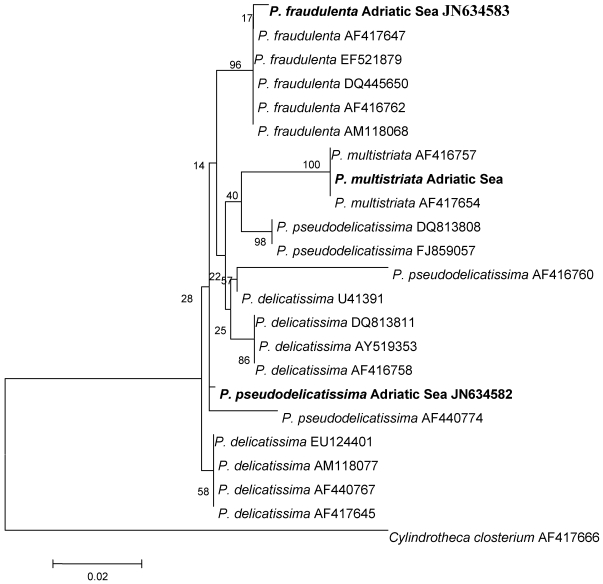
Phylogeny of *Pseudo-nitzschia* species inferred from sequencing the D1 and part of the D2 domains of 28S rDNA. Phylogeny was obtained from a Kimura-2-parameter model using Neighbor Joining reconstruction; the bootstrap support has been calculated for 1000 replicates. Strains sequenced in this work are in bold.

Following the occasional detection of low levels of DA in shellfish, a toxicity screening on different *Pseudo-nitzschia* species isolated from the Adriatic Sea was started in 2006. During the 2006 and 2007 seawater monitoring activities, several *Pseudo-nitzschia* species were also detected at bloom levels. Samples obtained through net hauls were used for algal isolation and culturing. However, of the total, only three strains could be successfully maintained in culture. At first, the three strains were tentatively identified on the basis of their morphometric characteristics [83,84] (Table 1) as *P. pseudodelicatissima*, *P. fraudulenta* and *P. multistriata.* Subsequently, their 28S rDNA sequences (about 400 bp corresponding to D1 and part of D2 domains) were obtained and analyzed ([85], for methods see [86]). The sequences were compared with those of strains present in GenBank and the resulting phylogenetic tree is shown in Figure 3. 

**Table 1:** Dimensions of *Pseudo-nitzschia* species isolated in Adriatic coastal waters (Studied strains) compared with published values (Ref. value).

	Apical Axis (μm)		Transapical Axis (μm)		Overlap of Cell Length	
	Studied Strain	Ref. Value	Studied Strain	Ref. Value	Studied Strain	Ref. Value
*P. pseudodelicatissima*	64.2–70.0	50–140 *^a^*	2.1–2.9	1.5–3.4 *^a^*	1/6	1/5–1/6 *^a^*
*P. fraudulenta*	75.6–80.0	50–119 *^a^*	5.0–6.7	4.5–10 *^a^*	1/5	/
*P. multistriata*	40.6–42.8	38–65 *^b^*	3.0–3.3	2.5–4 *^b^*	1/8	/

*^a^* [83]; *^b^* [84].

All of the *P. fraudulenta* and *P. multistriata* strains from different geographical areas, including the Adriatic strains, clustered together, and this result matched with the identification performed on morphometric basis. On the other hand, the Adriatic *P. pseudodelicatissima* strain clustered with both *P. delicatissima* and *P. pseudodelicatissima* strains leading to an uncertain phylogenetic origin. Therefore, it is not possible to rely only on results based on LSU sequences to definitely discriminate between the two species, as already reported in literature [87,88].

With regard to their toxicity, the three species were all considered potentially toxic although, as frequently observed in the genus *Pseudo-nitzschia*, both toxic and non-toxic populations were reported [89]. In particular, a *P. multistriata* strain from Italian waters (Napoli, Tyrrhenian Sea) was previously found to produce DA [87,90] as opposed to New Zealand strains in which the toxin was not found [91]. Toxicity analyses of the three strains from the Adriatic Sea were performed in cells collected in the advanced stationary phase after growing them at 20 °C under control (f/2) and P-depletion (f/2 medium with 1/10 P) conditions and, for *P. multistriata*, also under different temperatures (16, 20 and 25 °C, in nutrient replete conditions). *P. delicatissima* gave the highest cell yield (in the order of 10^6^ cell/mL) while the other two species reached cell numbers of nearly one order of magnitude lower. With regard to the effect of temperature on *P. multistriata* growth, a 40% lower cell yield was obtained in cultures at 16 °C with respect to those at 20 °C and 25 °C. *P. delicatissima* and *P. fraudulenta* did not contain DA, while *P. multistriata* was found to produce DA at low levels, comparable to those found in strains from Napoli coastal area, and amounting to 0.003 pg/cell. The algae grown at 20 °C and 25 °C, and under P-deficiency had similar toxin content, while in cultures kept at 16 °C, DA content significantly increased up to 0.010 pg/cell (ANOVA, *p* < 0.001; [92]). This is the first report on DA production by a cultured *Pseudo-nitzschia* species isolated from the Adriatic Sea which can be correlated to DA detection in farmed mussels; interestingly, in the field, *P. multistriata* cells reached the highest concentration (about 10^5^ cell/L) at higher temperature (*i.e.*, July–August 2007 according to growth results) and traces of domoic acid were found in mussels, afterwards, in November of the same year. However, the presence of other domoic acid producing *Pseudo-nitzschia* spp. cannot be excluded as different potentially toxic species have recently been detected in the Adriatic Sea [9,28].

### 4.6. Palytoxin Group

Since the appearance of toxic *Ostreopsis* spp. blooms, efforts have been focused on understanding the frame of environmental conditions that favor the onset and the development of this benthic dinoflagellate and its consequences on the ecosystem. Therefore, many studies either ecological or related to toxicity have been undertaken. *Ostreopsis* bloom development and toxic effects showed differences among the coastal areas facing the Adriatic Sea. Particularly, in the Northern Adriatic Sea, regions characterized mostly by sandy coastal waters were not affected (e.g., Veneto and Emilia-Romagna). Our studies have been mostly focused on several Adriatic and Tyrrhenian cultured strains with particular attention to the Northern Adriatic Sea (Marche region) [93,94]. At first, a study conducted on cultured strains isolated from the Adriatic and Tyrrhenian Seas [95] showed the presence of ovatoxin-a (OVTX-a) as the main toxin, and putative palytoxin (pPLTX) as the minor component. Further investigations highlighted the presence of four additional ovatoxins (OVTX-b, c, d, e) produced by *O.* cf. *ovata* [96]. The presence of the six analogs was confirmed by successive analyses in a total of four strains from the Adriatic and Tyrrhenian Seas and, generally, ovatoxin-a was by far the major component of the toxin profile (52–54% of the total toxin content), followed by ovatoxin-b (25–26%), ovatoxin-d and -e (13–15%), ovatoxin-c (5–6%) and putative palytoxin (1–2%). In these strains total toxin content ranged between 20 and 130 pg/cell; however, analyses of additional isolates are in progress.

### 4.7. Ichthyotoxins

The toxicity of the *F. japonica* strains (Raphidophyceae) associated with recurrent blooms in the Adriatic Sea was evaluated by considering all of the compounds previously postulated to be toxic to understand their role in toxicity of blooms [31]. Subsequently, further investigations have been performed on Adriatic *H. akashiwo* and *C.* cf. *subsalsa* isolates ([97] and below reported results). Several hypotheses have been, in fact, proposed for the toxic mechanism of the Raphidophyceae, and have included the production of mucus or other lectin-like polysaccharides that may cause asphyxiation by covering fish gills [98], the production of hemolytic compounds such as polyunsaturated fatty acids (PUFAs) [99] or reactive oxygen species (ROS) which damage fish gill tissue reducing oxygen uptake [100,101]; finally they have also been claimed to produce a toxin similar in structure to brevetoxins [102].

The cellular fatty acid profile of the Adriatic *F. japonica* evidenced the presence of a high amount of PUFAs, including the three already demonstrated as hemolytic, such as 18:4n-3 (stearidonic acid, OTA), 20:4n-6 (arachidonic acid, AA), and 20:5n-3 (eicosapentaenoic acid, EPA). Fatty acid composition and quantification of this Adriatic strain showed values in accordance with those reported for Japanese and New Zealand strains (summarized in [31]), except for a lower amount of AA (20:4n-6). LC-MS analyses of the cultured strain excluded the presence of the known brevetoxin, BTX2. Several toxicological assays showed rather different results likely due also to the different organism used in the bioassay: the effect of *F. japonica* cell extracts on *Vibrio fischeri* bioluminescence was high, while mortality of *Artemia* nauplii occurred only when organisms were exposed to high cell concentrations (long-term assay), probably leading to accumulation of a high amount of fatty acid per single organism; the hemolytic assay with *Cyprinus carpio* erythrocytes showed that increasing cell concentrations of *F. japonica* caused hemolysis of up to 100% erythrocytes. This result could be indicative of a marked ichthyotoxic potency of this species; however, the fish assays evidenced a rather delayed effect of the Adriatic *F. japonica*, with mortality occurring only after 9–10 days of exposure. The significant increase of hydrogen peroxide (H_2_O_2_) obtained in the tanks where sea bass were exposed to *F. japonica* indicated that the presence of fish stimulated H_2_O_2_ production by algal cells and supported the hypothesis of H_2_O_2_ involvement in its toxicity [31], as already suggested for *Chattonella marina* [101,103]. Moreover, the presence of cellular PUFAs in high amounts could substantiate the hypothesis of a combined effect of these compounds and ROS. 

Subsequently, the presence of some of the known BTXs was investigated more deeply in the Adriatic Raphydophyceae *F. japonica*, *H. akashiwo* and *C.* cf. *subsalsa* by LC-MS/MS analyses, to check for the presence of some of the most common brevetoxins (BTX1, BTX2 and BTX3) and their metabolites (BTX-B2 and BTX-B5). None of these toxins was found in these strains, leading to the conclusion that they do not produce any of these known brevetoxins [104]. Several bioassays have been also performed to investigate the toxicity of *H. akashiwo* and *C.* cf. *subsalsa* ([97], for methods see [31]). Results showed that after 24 h (short-term assay) EC_50_ values calculated on the basis of the observed nauplii immobilization (Table 2) were 3671 and 485 cell/mL for *H. akashiwo* and *C. subsalsa*, respectively, leading to a higher toxicity for *Artemia* of these two species with respect to *F. japonica* (EC_50_ value: 1.5 × 10^5^ cell/mL) [31].

**Table 2:** The 50% effect concentrations (EC_50_) of *Heterosigma akashiwo* and *Chattonella* cf. *subsalsa* obtained in the *Artemia* sp. short-term assay and in the hemolytic assay with *Cyprinus carpio* erythrocytes. The reported values are means ± SE of three replicates.

	EC_50_ (cell/mL)	
	*Artemia* sp. Assay	Erythrocyte Lysis Assay
*Heterosigma akashiwo*	3671 ± 59	187,263 ± 7152
*Chattonella* cf. *subsalsa*	495 ± 15	27,161 ± 8031

Erythrocyte lysis assay (ELA) was carried out with *C. carpio* erythrocytes: *H. akashiwo* and *C.* cf. *subsalsa* showed low hemolytic effects, and resulted in very high EC_50_ values (1.9 × 10^5^ and 2.7 × 10^4^ cell/mL, respectively) corresponding to cell abundances never detected in the environment for these species (Table 2). *F. japonica* was the species with the highest hemolytic activity, as evidenced by the lowest EC_50_ value (5.2 × 10^3^ cell/mL) [31]. The fish assay reported a delayed ichthyotoxic effect of *H. akashiwo* and *C.* cf. *subsalsa*, with mortality occurring only after 10 and 14 days of exposure, respectively. Thus, overall the Adriatic Raphidophyceae strains showed low ichthyotoxicity when compared to the high ichthyotoxic potency of Japanese strains; it seems in fact that a high cell density and a long exposition time are necessary to cause severe damage on the fish gills or death for crustaceans, thus justifying the absence of fish kill events during the frequent and dense bloom episodes along the Italian coasts [31].

## 5. Conclusions

This review is the first to report on: (1): the toxic molecules found in shellfish from the Adriatic Sea; (2): the algal species involved in their production; and (3): studies performed to assess toxicity of potentially toxic algae blooming in this area (summarized in Table 3). Data so far collected and reported herein on Adriatic harmful algal species reveal a unique toxicological profile of this Italian Sea. Moreover, the Adriatic toxicological scenario appears to be in continuous evolution in terms of predominant species and, accordingly, of toxins production. Furthermore, farmed shellfish often present a complex toxin spectrum due to the occasional detection of ever new toxic molecules of unknown origin [16,105,106,107,108,109].

**Table 3:** Phytoplankton species reported in the present study, their toxins profile and levels detected in cultures; related symptoms and periods either of toxin detection in Italian farmed mussels or of the algal species presence in coastal waters are also reported.

Species	Toxin	Toxin Levels	Effect	Toxin Presence in Mussels (Year)
*Dinophysis* spp.	OA, DTXs	n.d.	DSP	1989–1997, 2010
*Protoceratium reticulatum*	YTX prevalent, homoYTX, 45-OHYTX, carboxyYTX, noroxoYTX	4.5–65 pg/cell (YTX)	Neurotoxic through i.p. injection	1995–2006
*Lingulodinium polyedrum*	Homo-YTX	from none to 0.80 pg/cell	Neurotoxic through i.p. injection	1996
*Gonyaulax spinifera*	Homo-YTX prevalent, YTX	33,4 pg/cell (Homo-YTX)	Neurotoxic through i.p. injection	2007
*Alexandrium minutum*	GTX4 prevalent, GTX2, GTX3, GTX1	964 fg/cell (GTX4)	PSP	1993–1994
*Prorocentrum lima*	OA prevalent, DTX1, DTX2, DTX4, DTX6	6.7–8.4 pg/cell (OA)	DSP	2002
*Alexandrium ostenfeldii*	13-desMe C, 13,19-didesMe C e 27-OH-13,19-didesMe C prevalent plusspirolides A,B,C,D, 13-desMe D	3,7 pg/cell (13-desMeC)	Neurotoxic through i.p. injection	2003
*Pseudo-nitzschia multistriata*	Domoic acid	0.003–0.010 pg/cell	ASP	Sporadic since 2000
				**Bloom Presence**
*Ostreopsis* cf. *ovata*	Ovatoxin-a prevalent, OVTX-b, c, d-e, pPLTX	20–130 pg/cell (total)	Respiratory distress	Recurrent since 2001
*Fibrocapsa japonica*	ROS, PUFA	-	Ichthyotoxic	Recurrent since 1994

n.d. = not determined

Toxic phytoplankton poses great threats to human health; therefore, many efforts need to be addressed in shellfish chemical analysis with the ultimate goal of assessing whether their commercialization can be allowed. A further step towards comprehension of toxic events can be represented by studies performed on cultured microalgae and, although the necessary information is obtained with a certain delay, they can be of great help in the management and prevention of the toxic outbreaks. 

The results presented in this review highlight the importance of cultured toxic species for studies concerned with harmful algal blooms in the Adriatic Sea. Firstly, when new toxins are detected in mussels, the analysis of even one cultured isolate is important for identification/confirmation of the causative organism. Secondly, by analyzing a certain number of isolates, the characterization of the whole toxic profile can be achieved, thus being of help for mussel monitoring. In addition, the comparison among toxic and genetic profiles of strains from different geographical areas is useful for assessing environmental algal distribution due to natural (e.g., marine currents) and/or anthropogenic (e.g., ballast waters) dynamics. Comparison among different strains of the same species is also important for evaluating the role of geographical isolation and/or environmental conditions on toxin gene expression. Finally, the cultured biomass can provide reference material both for chemical analysis, especially when new toxins are detected, and for further studies on toxin mechanism of action and toxicology. 
